# Flexible knowledge repertoires: communication by leaders in trauma teams

**DOI:** 10.1186/1757-7241-20-44

**Published:** 2012-07-02

**Authors:** Maritha Jacobsson, Maria Hargestam, Magnus Hultin, Christine Brulin

**Affiliations:** 1 Department of Social Work, Umeå University, Umeå S-90187, Sweden; 2 Department of Nursing, Umeå University, Umeå S-90187, Sweden; 3 Department of Surgical and Perioperative Sciences, Umeå University, Umeå S-90187, Sweden

**Keywords:** Leadership, Communication, Teamwork, Simulation, Discourse psychology

## Abstract

**Background:**

In emergency situations, it is important for the trauma team to efficiently communicate their observations and assessments. One common communication strategy is “closed-loop communication”, which can be described as a transmission model in which feedback is of great importance. The role of the leader is to create a shared goal in order to achieve consensus in the work for the safety of the patient. The purpose of this study was to analyze how formal leaders communicate knowledge, create consensus, and position themselves in relation to others in the team.

**Methods:**

Sixteen trauma teams were audio- and video-recorded during high fidelity training in an emergency department. Each team consisted of six members: one surgeon or emergency physician (the designated team leader), one anaesthesiologist, one nurse anaesthetist, one enrolled nurse from the theatre ward, one registered nurse and one enrolled nurse from the emergency department (ED). The communication was transcribed and analyzed, inspired by discourse psychology and Strauss’ concept of “negotiated order”. The data were organized and coded in NVivo 9.

**Results:**

The findings suggest that leaders use coercive, educational, discussing and negotiating strategies to work things through. The leaders in this study used different repertoires to convey their knowledge to the team, in order to create a common goal of the priorities of the work. Changes in repertoires were dependent on the urgency of the situation and the interaction between team members. When using these repertoires, the leaders positioned themselves in different ways, either on an authoritarian or a more egalitarian level.

**Conclusion:**

This study indicates that communication in trauma teams is complex and consists of more than just transferring messages quickly. It also concerns what the leaders express, and even more importantly, how they speak to and involve other team members.

## Introduction

In the emergency care of injured people, the goal is to save the patient’s life. In order for the emergency team to achieve the best outcome, it is extremely important to communicate observations and assessments [1]. To improve communication in emergency care, communication strategies developed in the military have been adapted to streamline the linguistic interaction. One of the strategies used to improve communication within a team is “closed-loop communication”; this can be described as a transmission model in which feedback is of great importance. When using closed-loop communication, the leader makes the team members aware of important observations by asking questions about the patient’s condition. The question is addressed to one of the team members, who then has to show that he or she is paying attention by giving feedback to the leader [2]. The leader then closes the loop by confirming that the message has been correctly understood. The purpose of this closed-loop communication is to enhance communication efficiency and minimize misunderstandings. For example, studies from obstetric emergencies have found that teams using closed-loop communication were more efficient in managing critical tasks compared to teams with less clear communication style [3].

Crisis Resource Management (CRM) is a program aimed at creating an expert group to act and think as a team, working towards a collective goal [4]. Team members are trained to support the leader, but they also have a responsibility to speak up when they feel the leader is making the wrong decision [5,6]. Studies from the aviation industry have shown better outcomes in collaboration and communication when attitudes improved among the personnel in the cockpit [7]. Similar experiences have also been found in health care [8-10]. Closed-loop communication and CRM can both be seen as consensus-seeking processes in emergency teams, but the roles of the leaders differ to some extent. In closed-loop communication, the leader occupies a clear, authoritative role, while in the CRM program leaders are encouraged to listen more to team members. One problem with these approaches is that they were developed to suit a military context [7]. The environment in medical emergency situations is different, in that the focus is on a patient’s survival [11]. It is therefore important to further evaluate these models in a medical emergency situation, since they have previously been evaluated only sparsely in that context. In this study, we focused on how formal leaders communicate knowledge, create consensus, and position themselves in relation to others in the team.

## Background

Teamwork is a common and traditional form of collaboration within health care, but the structure of teams varies. For instance, some teams include highly skilled and specialized individuals from the same profession, while others include individuals from various professions with a shared task and goal [12]. This latter constellation can be described as an interdisciplinary team, in which different professions work together in a clinical context with a willingness to acknowledge the roles, competencies, and responsibilities of other professionals [13].

### Leadership

The leadership role is often described as a core component in the team and has a considerable impact on the team and its efficiency [14]. The team needs a leader who takes command in the team, initiates the progress of the work, and coordinates the members’ performance systematically [5,8,15,16]. In health care, knowledge is strongly linked to power (cf. [17]), and power in this context is further linked to the profession. In emergency praxis, the designated formal leader in the team is often a physician (surgeon) who is considered to have expert knowledge [18]. Physicians usually work quite independently [19] with little or no awareness of team members’ skills and knowledge. These circumstances are probably key factors, and it is a challenge for the formal leader to coordinate team members’ tasks in order to optimize teamwork to obtain a successful outcome.

The tasks carried out by emergency interdisciplinary teams are complex, unpredictable, and urgent. Team members work under time pressure [16,20], and the composition of the team changes depending on which personnel are currently on call. It may therefore be a challenge to achieve consensus in order to accomplish the task efficiently. Consensus processes require “some shared understanding and common commitment” [21] of the common goal of the teamwork, and in relation to strategic issues.

### Studying leaders’ repertoires

In studying leaders’ repertoires, we were inspired by discourse psychology [22] as well as Strauss’ concept of “negotiated order” [23]. According to Potter and Wetherell, language is not just a mirror of reality; in fact, reality is constructed by language [22]. The ways in which we communicate should therefore be studied in the context where they are developed. We used the analytical concept of “interpretative repertoires” [24,25] to capture how leaders strategically choose repertoires and position themselves in order to communicate knowledge and achieve consensus in the teams. We analyzed both what they were saying, and how they expressed themselves. Strauss [23] implies that all social order is negotiated. In all groups or organizations that work together, negotiations between individuals are required to “work things out”. In other words, communication within an organization is not concerned merely with the transfer of orders. Collaboration does not always work out; and even when it does, effort is required to maintain it. Strauss describes different negotiating strategies that can be used to achieve consensus. Different organizations tend to use certain strategies, such as negotiation, education, coercion, and discussion [26]. These strategies may be compared with so-called discursive strategies: bonding, encouraging, directing, modulating, and re/committing [27]. In terms of interpretative repertoires, we can talk about negotiating, educational, coercive, and discussing repertoires. The repertoires are often flexible [24,25] meaning that the leader can take different positions and use different repertoires in the interaction [28].

Building on this empirical challenge and theoretical base, the purpose of this study was to analyze how formal leaders communicate knowledge, create consensus, and position themselves in relation to others in the team. We also discuss in this paper the consequences of the leader’s performance, for the team and for the health of the patient.

## Material and method

This project was a collaboration between Umeå University (including the Departments of Education, Nursing, Social Work, and Surgical and Preoperative Sciences), Västerbotten County Council (VLL), the Swedish Defence Research Agency (FOI), and Nordic Safety and Security (NSS).

### Participants

The participants consisted of personnel in trauma team training at a hospital in northern Sweden. The personnel comprised a total of 19 teams, from which one team was excluded due to illness and two were excluded due to technical problems. Each team consisted of one surgeon or emergency physician (designated team leader), one anaesthesiologist, one nurse anaesthetist, one enrolled nurse from the theatre ward, one registered nurse and one enrolled nurse from the emergency department (ED) - a total of six participants per team. Thus, a total of 96 participants were included in the study (physicians n = 32, registered nurses n = 32, enrolled nurses n = 32). In Table 1 characteristic of the team leaders are presented.

**Table 1:** Characteristics of trauma team leaders

	
Age (years), (means ± SD)	41.1 ± 11.7
Years in profession, (means ± SD)	10.2 ± 11.2
ATLS certified, n (%)	15 (94%)
Male, n (%)	11 (69%)

Participants were informed that the recorded material would be handled confidentially, that no-one outside the research group would have access to the coded audio and video material, and that no individual would be identifiable in the reports. The material from the audio and videotapes was stored and archived in a place inaccessible to unauthorized personnel. Informed consent was obtained after written information was given to all participants. The study was approved by the Regional Ethical Review Board in Umeå (9 June 2009, Dnr 09-106 M).

### Research setting

The trauma team was audio- and video-recorded during high fidelity simulation training in a hospital in northern Sweden. To increase the authenticity of the resuscitation, the participants performed normal tasks in their own roles in the standard emergency room (ER) in the ED with standard equipment and protocols. The “patient” was an advanced human patient simulator (HPS), (SimMan 3 G, Laerdal Medical, Stavanger, Norway). The HPS was pre-programmed to represent a severely injured patient suffering from hypovolemia due to external trauma. Before the training, the participants were asked to view an introductory video about teamwork in emergency settings, including theoretical discussion with a focus on leadership and communication. They were also encouraged to act as authentically as possible, and the environment and timeframes were explained.

The training session started with the ED nurse alerting the trauma team about a trauma incident. The trauma team was summoned to the emergency room at the ED and informed about the case incident by the ED nurse. The team members then introduced themselves to each other and started to prepare the emergency room with equipment and materials according to the hospital’s standard operating procedures for trauma care. When the patient arrived at the ED, the initial assessment and actions began. Assessment of the HPS was based on current guidelines in the hospital and was performed systematically according to the Advanced Trauma Life Support program [29] with the goal being early identification of the patient’s injuries. Each simulation scenario was designed to last for 15 min before the instructor interrupted the session. The participants were asked not to disclose the patient scenarios to their colleagues outside the room. Before the session began, the instructors reinforced the principle of discretion about the team’s and the individual team members’ performance.

### Data collection

Data were collected from November 2009 to March 2010. Video recording was performed using standard video surveillance cameras. Three video cameras were placed in the emergency room and one in the office where the ED nurse received the alarm. Individual wireless microphones registered the communications of each of the team members. All data were collected in F-Rex, a software program developed by the FOI (Swedish Defence Research Agency, Linkoping, Sweden), to allow reconstruction and investigation of an incident. Observations during the team training were made and field notes were taken by one of the authors (MH).

### Data analysis and method

The videos were analyzed by the first two authors (MH, MJ), and the communication component of the audio-recorded material was transcribed verbatim by MH. MH and MJ each read through the transcript independently. Material from five of the teams was analyzed in depth and was selected due to the good quality of the audio. When transcribing the material, the communication between the actors in the teams was categorized into “turn-constructional units” according to conversation analysis [30,31]. By detailed reading, flexible interpretative repertoires were identified in line with Corbin & Strauss’ (1993) concepts; coercive, educational, discussing, and negotiating. Another category identified was communication failure. The data were then organized and coded using the qualitative data analysis software program NVivo 9 [32]. This approach was chosen in order to highlight how flexibly the formal leader used interpretative repertoires and how they changed their position in the team [33]. In the analysis, we mainly focused on how the formal leader communicated as a leader with the team members.

## Results

Most of the repertoires were initiated by the leader and addressed to the anaesthesiologist or to one of the nurses. The leaders were flexible, using coercive, educational, discussing, and negotiating repertoires in order to obtain knowledge and control of the situation. In some cases, they failed to achieve consensus: for example, when the leader was ignored and none of the members in the team were listening. In Table 2, the utterances are quantitatively categorized into different repertoires. As illustrated, use of the different repertoires varied both between and within the teams.

**Table 2:** Frequencies of strategies coded into turn-constructional units

**Strategy**	**Coercive**	**Educational**	**Discussing**	**Negotiating**	**Working**
**Team**	**repertoire**	**repertoire**	**repertoire**	**repertoire**	**failure**
Team A	10	10	12	0	0
Team B	1	1	2	4	6
Team C	7	0	5	3	1
Team D	0	0	0	2	6
Team E	4	2	5	11	2
Team F	0	0	9	0	0
Team H	4	1	13	0	0
Team J	5	4	7	4	1
Team K	1	2	7	0	1
Team L	2	0	2	0	1
Team M	0	0	2	1	1
Team N	2	2	2	1	1
Team O	1	0	3	2	2
Team P	5	0	3	1	0
Team R	3	0	4	1	0
Team S	3	2	5	1	3
**Total**	48	24	81	31	25

To give a deeper understanding of how the leaders used these repertoires, we will give some examples from the material. These examples are selected because they are coherent excerpts that contextualize and illustrate the various strategies. The names of the participants in the examples are modified in order not to reveal their identities. The following abbreviations are used: “L” (leader), “An” (anaesthesiologist), “NurseED” (registered nurse from the emergency department), “NurseAn” (nurse anaesthetist), “EnrolledAn” (enrolled nurse from the theatre ward), and “Instr” (the instructor for the scenario).

### Coercive repertoire

By using coercive repertoire, the leaders positioned themselves as superior, with the aim of coercing team members into listening to and acting on their decisions. To create consensus, the leader gave orders, asked closed or leading questions, and expected only short responses from team members. The care of the HPS followed the principles of a strict A, B, C, D, E ordering, where the airway (A) was the first priority. In our study, this repertoire was only used during urgent situations. In the following excerpt, the patient has just arrived in the ER and the leader initiates the assessment of the patient:

L: So, first priority remains the airway.

L: We’re working on it.

L: What is the … yes?

An: Do we have breath sounds?

NurseAn: Pulse oximeter on.

L: I’ll listen [uses stethoscope].

Instr: 60 in saturation.

An: I’ll put the mask on and a bag so I can ventilate.

NurseAn: OK.

L: OK, can we work on the airways?

An: Yes, coming here.

NurseED: I’ll start the infusion line.

An: Then we ventilate.

L: I hear no breath sounds either on right or left side.

L: The airway still enforced?

As illustrated in this excerpt, the leader selected and controlled the topic by using a coercive repertoire, with “airway” (A) being the central topic; he expressed knowledge and experience of these important principles. The leader had a direct conversational style [34] and a clear way of expressing his thoughts, using the phrases “first priority” and “work on the airway” and suggesting that the members should perform certain tasks. The leader made exhortations to the members, which would imply that the leader was in control of the situation. The leader gave no sign that he intended to listen to the other members of the team.

When leaders used coercive repertoire, they gave the impression that they alone had the knowledge needed, and that they were in control of the communication flow.

### Educational repertoire

The second type of repertoire had a more educational style. As in the previous example, leaders transmitted their knowledge to the other members in the team, but the difference was that leaders using this repertoire gave the impression that they wanted to make the members understand why it was important to prioritize in a certain way. The leaders again positioned themselves on a superior level and communicated in a direct conversational style. These repertoire gave the impression that, as leaders, they possessed key knowledge and were willing to share this knowledge. In comparison with those using coercive repertoire, leaders using an educational repertoire seemed to be more willing to listen to team members. In the following excerpt, the leader communicates with team members, using an educational repertoire just before the patient arrives at the ED.

L: OK, penetrating trauma, ehh, the roles are clear, yes, the ones we usually have.

NurseED: Yes.

L: So as usual … the anaesthesiologist’s primary task is the airway, fluids.

NurseED: Eva, please take down the yellow gowns.

L: Undressing is very important in this kind of patient, so that we get it done quickly, then you take samples (eye contact with the NurseED).

L: And then, ehh, start fluids early, the goal with these patients must also be that we very quickly get to the logroll, very quickly so that we have all the injuries clear to us, both front and back of the patient much earlier than in a patient with blunt trauma, so that’s the only thing that is different when we know there is a knife injury.

The leader in this case used small pauses and sought eye contact to ensure that the members in the team were listening. This could be a strategy for the leader to emphasize certain considerations. At the same time, team members provided backup to the leader in the form of small nods, indicating their support for the leader’s continuing to talk. In this repertoire, the leader expressed opinions about the team’s priorities, and in the excerpt the leader strengthened this by using the adverb “very”. The leader clarified roles and prioritized tasks within the team, using distinct language when clarifying the priority of the tasks that all members in the team should be involved in. The leader clarified the roles and tasks by asking a rhetorical question - “roles are clear, yes” - which could be considered as a statement that appears to involve the other team members. With phrases such as “very important” and “quickly”, the leader reinforced the message and alerted the team to the actions which were important to concentrate on. By repeatedly referring to “we”, the leader expressed a sense of consensus in the team.

When leaders used educational repertoire, as with coercive repertoire they again gave the impression that they alone had the knowledge that was needed. The difference with educational repertoire was that in such cases they seemed to be intending to improve team members’ own knowledge.

### Discussing repertoire

When leaders used discussing repertoire, they positioned themselves on a more interactive and equal level. With these repertoire, they gave the impression that they had found a common understanding of the situation by involving the other team members in the decision. Together with the team members, the leaders discussed possible explanations of the patients’ symptoms. In the example below, this leader seems to expect a response from the recipient:

L: It sounds unlikely that the patient would be unconscious of … then he would have bled … it sounds a bit unlikely, but he might have other injuries, you should almost suspect or believe that.

An: What did you say?

L: Also, it sounds a bit unlikely that there’s only one stab injury in the left arm.

An: Yes, it is a bit.

L: If he is unconscious or seriously…

An: Yes, perhaps the patient has lost a lot of blood, has lost …

In this excerpt, the leader initiated a discussion and invited the anaesthesiologist to join in the decision making by asking the anaesthesiologist’s opinion about the patient’s condition. The question was directed to a specific person - “you” - and consensus about the task was achieved by involving the team members. In this case, the leader was using a high involvement style [34], showing commitment to other members and sharing decisions within the team. The leader also gave an impression of uncertainty by using vague expressions, such as “sounds a bit unlikely” and “you can almost suspect or believe”. This gave other team members the opportunity to comment on the leader’s utterances. The leader encouraged the members and involved them in the decision making, listening to their opinion.

### Negotiating repertoire

The negotiating repertoire were similar to the discussing repertoires. However, in the negotiating repertoires, leaders encountered more resistance from team members and had to do more negotiating of knowledge. In the following example, the anaesthesiologist takes control of the work and decides the priority for the care of the patient:

L: We have a C problem and the heart’s beating.

An: But no palpable pulse.

L: No palpable pulse. What about the ECG? Did we get together something so we can obtain frequencies, or are there…

An: Do we have another needle, another one, Anna?

L: Mmm, Anna, we need another IV catheter, another one.

NurseED: On this wounded arm? Which is…

An: Insert, do we have … insert one on the leg then, so we have…

L: Mmm.

NurseAn: Can I insert an IV catheter here, on the right arm?

Instr: On the left arm, you can put it there.

L: We insert the IV catheter on the left.

An: Put another IV catheter on the left, OK.

An: Hana, we … it’s a knife wound, we have to turn the patient right away.

L: Yes, I’m aware of that.

L: I thought, I thought we have a C problem; I just want to get IV catheters in, before we turn.

An: IV catheters first.

An: Yes.

L: Just fix it because we have a big C problem here.

An: Yes.

L: And we have blood here as well, so we just have to get IV catheters first, ECG?

NurseAn: Shall I check the blood?

In this excerpt, the leader gave the impression of listening to team members and was open to discussion, which could be advantageous if the intention was to reach a common consensus. However, the leader in this case seemed not to rely solely on personal ability, but sought support and knowledge from within the team. The leader repeated the priorities suggested by the anaesthesiologist and expressed uncertainty by using terms such as “I thought”. A negotiation arose when the anaesthesiologist suggested a logroll, and the leader had a different opinion on what should be prioritized. The leader first justified an opinion, and then regained control over the leadership; the anaesthesiologist withdrew by saying “yes”, “OK”.

When leaders used this repertoire, knowledge was negotiated within the teams, and the leaders showed a higher degree of uncertainty of their own knowledge.

### Working failure

Sometimes, none of the repertoires previously presented seemed to work; the leader was ignored by team members and not listened to, which led to their being in a subordinate position. One example is presented below. In this case, the formal leader did not step forward and take the leadership role, but instead gave an impression of being uncomfortable with that role, for instance by talking almost in a whisper. In the following two excerpts, the leader wants to assess the patients’ back before doing X-ray, computed tomography (CT):

L: He’s remaining stable, so let’s turn him and then we’ll go to traumaCT then.

An: Yes, okay.

An: Have you put … have you asked for some pictures under him?

L: No, we haven’t done that.

An: No, okay.

An: So we go on to traumaCT then.

An: Should we put the catheter in?

L: Yes, absolutely! We’ll put it in, so I can peek in underneath but there’s nothing bleeding externally or anything…

An: The arm, is there something bleeding?

L: No, that also …

An: Hm.

L: Stable, so we’ll take a peek at that

An: Yes please … good that you’re bringing a catheter, Eve.

In that excerpt, the anaesthesiologist changed the subject and the leader changed the priorities. In the next excerpt, the leader tries to regain control and suggests checking the patient’s back:

L: And check the back, then to the trauma CT.

NurseED: Yes, but exactly…

An: Temperature?

An: Do we have his temperature?

NurseAn: No.

Instr: 34 and 2.

An: Temperature 34 and 2, thanks, then he hasn’t dropped.

An: It is higher.

An: Can I have a … Helen…

EnrolledAn: Yes.

An: From you … can I have a urinary catheter bag from one of you, ehh, up here?

An: I have no uribag up here as far as I can see.

In both of these examples, the leader suggested a logroll. The anaesthesiologist seemed not to be listening and changed the topic, gave no recognition to the leader’s priority, and continued to control the choice of topic. The anaesthesiologist avoided eye contact with the leader, turned away from the leader, and communicated directly with other team members. Thus, the formal leader was systematically excluded from the communication by the anaesthesiologist, who took over the leadership role. Despite this takeover, the leader tried to control the priority of the assessment but failed because none of the members in the team was listening. The leader also tried to reiterate the suggestion for logroll and checking the patient’s back, a total of three times during the scenario. It should be noted that a leader being positioned on a subordinate level does not necessarily mean that the work in the team will collapse, but it may delay the assessment and lower the efficiency (cf. [27]).

## Discussion

The findings suggest that communication between the leaders and other team members was flexible and cannot be described simply as a transmission model in line with the “closed- loop” model. In this study, leaders used different repertoires to convey their knowledge to the team, in order to create a common goal of the priorities of the work. One interpretation we can make is that the changes in repertoire were dependent on the urgency of the situation and that they performed different functions in the interaction. Our results also indicate that a leader’s position in a team may vary depending on the repertoire and the interaction. A study of leaders in business organizations [27] found that leaders used different strategies to build consensus in their teams, and the authors pointed out that the choice of strategies would probably affect the outcome of the consensus building. Wodak et al. also found two distinct leadership styles: an egalitarian style of leadership and an authoritarian style [27]. Leaders used different strategies in order to persuade, encourage, involve employees, and invite members into the discussion. The best way to achieve durable consensus in the team was to keep a good balance between different leadership styles, but with an emphasis on the authoritarian style [27].

We also found these leadership styles in the present study. When the leaders acted with a more authoritarian style, they used coercive and/or educational repertoires. In these situations the leader took a superior position, meaning that the relationship was unequal. Team members were silent, mainly listening, and did not question leaders’ knowledge and priorities. This could be explained in situations where the leader had a strong ethos and expressed competence. Leaders’ knowledge and technical competence seemed to instil confidence within the team, a result also reported by Hjortdahl, Ringen, Naess and Wisborg [20] whose study participants believed that a leader could not be a good leader without being an experienced surgeon. This hierarchical communicating strategy may be relevant, given that it is the leader who has the key knowledge, though there is a risk that important knowledge may not be utilized since no one in the team is invited to speak up.

Power differences in an interdisciplinary team may inhibit members who wish to speak up [12]. Edmondson indicates that minimizing power and status differences in the team to facilitate communication is of importance for the leader [12].

Medical teams act in an institutional context and, within this discourse, the doctor is in authority. Mishler talks, for instance, about the “voice of medicine” (in relation to patients) and Måseide talks about the “doctor’s voice” [35,36]. The team talk is dominated by these “voices” which relate to the physician’s individual and direct knowledge, practice, and judgement. This is not necessarily a personal expression, but an exchange of information to present professional or collective opinions [36].

In our study, when leaders acted with a more egalitarian style, they used discussing and/or negotiating repertoires. These leaders were positioning themselves on a more equal level. This type of leadership is characterized by a leader who allows autonomy and space and invites team members to join the discussion. Måseide indicates that the decision making is a collaborative problem-solving system accomplished through talk where medical evidence ispresented [36]. Within this discourse physicians take their turns to talk only when called for or when their contributions are relevant or necessary for the topic; they do not interrupt, and talk ends with a conclusion [37].

One disadvantage of the negotiating repertoire is that the discussion can develop into a power struggle between the members in the team, which can influence the quality of the work, probably leading to delays and, in the worst case, to failure [26]. Wodak et al. [27] found that when team members were invited to join in the discussion, they felt encouraged by the leader to explore new ideas and/or develop existing ideas. There are limitations to this process in emergency care situations, since there is time pressure relating to the patient’s condition and little or no time for discussion.

One way to develop a better discussion climate in the teams could be to share the leadership. Klein, Ziegert, Knight, and Xiao [16] describe what they call a shared leadership, where the formal (often junior) leader is supported by a more senior leader. Depending on the patient’s condition and the actions of the formal leader, the senior leader can step back. A dynamic delegation between the formal leader and the senior leader may encourage junior leaders to develop their skills and knowledge. In our study, the less experienced leaders had no support from an attending senior leader, so when necessary the anaesthesiologist took over the leadership role and carried the work forward. In the study by Hjortdahl et al. [20], inexperienced leaders expressed the opinion that support and guidance from more experienced team members was important when they were worried about missing serious injuries.

In the present study, leaders sometimes used the repertoires successfully, but some of them failed to create a common goal within the team. However, the teamwork still seemed to function in the last excerpt quoted, probably because a more experienced anaesthesiologist took on the leadership role. Since the formal leader was ignored despite several efforts to convince the team to perform a logroll, it is possible that this created uncertainty in the group. This kind of problem could also cause delays in patient care and could impair team performance. It could be that a shared leadership would create greater security and prevent similar situations from occurring. We do not know why some of the leaders in the present study seemed to have more working failures than others, though it is possible that it had to do with the ethos of the physician. Hjortdahl et al. point out in their study that not all team leaders feel prepared for this task. Working failures occurred more often in teams led by women than in teams led by men, and in one of the “failing” teams, the leader also was a non- Scandinavian. Eklöf argues that during the twentieth century a picture of the “ideal physician” emerged in Sweden [38]. According to that picture the ideal physician has a strong ethos, which means a person with authority and a good character. Moreover, he has scientific knowledge and certain characteristics: he is a white middle-aged man with a good reputation and shows conscientiousness, humour, and personality. Some of these attributes have also been expressed by trauma team members asked to describe a good leader [20]. More specifically, the participants described a successful leader as a person with a high level of professional knowledge, who communicates clearly and distinctly with confidence and calmness, creating security within the team. The team members had to be able to trust their leader. However, inexperienced leaders expressed their insecurity in their own knowledge, the wrong decisions they made, and their own performance; they also expected more experienced team members to guide them [20]. This study pointed out problems in leaders’ communication repertoires that led to failures in teamwork. Therefore it is of great importance to further study associations between leaders’ communication and outcomes in high fidelity training in an emergency department while taking gender and ethnicity into consideration.

This study indicates that communication in trauma teams is complex and consists of more than just transferring messages quickly. It also concerns what leaders express, and even more importantly, how they speak to and involve other members in the team. Leaders in trauma teams should consider different knowledge repertoires and understand how leaders may position themselves in the interaction to facilitate conveying knowledge and achieving consensus during team performance. In a future study, it would be of interest to investigate communication in a trauma team during a real situation.

### Limitations of the study

Simulation training gives participants the opportunity to train for low frequency events in a high fidelity setting. In this study, the context was realistic as the training took place in the emergency room and the participants trained in their normal professionals roles within the trauma team. This should decrease the risk of poor performance due to lack of enthusiasm for this kind of simulation training. In addition, using HPS in an authentic environment provides the opportunity to standardize a trauma case scenario and compare the team’s performance, both from a team perspective and a patient outcome perspective. The various patterns of leadership under pressure that have been presented in this paper are also the ones most likely to be present in real life.

## Competing interests

The authors declare that they have no competing interests.

## Authors’ contributions

All authors were involved in the design of the study. Data were collected by MH and MHu and analyzed by MH and MJ. The results were fully discussed by all authors. All authors worked on the preparation of the manuscript and the final manuscript was approved by all authors.
